# Physician-led versus questionnaire-based psychosocial screening in adults with high-grade glioma: a cluster-randomized controlled trial (GLIOPT)

**DOI:** 10.1007/s11060-025-05223-6

**Published:** 2025-09-10

**Authors:** Mirjam Renovanz, Melina Hippler, Robert Kuchen, Lorenz Doerner, David Rieger, Joachim P. Steinbach, Michael W. Ronellenfitsch, Martin Voss, Almuth F. Kessler, Vera Nickl, Martin Misch, Julia Sophie Onken, Marion Rapp, Minou Nadji-Ohl, Marcus Mehlitz, Jürgen Meixensberger, Michael Karl Fehrenbach, Naureen Keric, Florian Ringel, Jan Coburger, Carolin Weiß Lucas, Jens Wehinger, Friederike Schmidt-Graf, Jens Gempt, Marcos Tatagiba, Ghazaleh Tabatabai, Melanie Schranz, Susanne Singer

**Affiliations:** 1 Department of Neurology & Interdisciplinary Neuro-Oncology, Hertie Institute for Clinical Brain Research, University Hospital Tübingen, Eberhard Karls University Tübingen, Tübingen, Germany; 2 Center for Neuro-Oncology, Comprehensive Cancer Center Tübingen-Stuttgart, University Hospital Tübingen, Tübingen, Germany; 3 Department of Neurosurgery, University Hospital Tübingen, Tübingen, Germany; 4 Institute of Medical Biostatistics, Epidemiology, and Informatics (IMBEI), University Medical Center Mainz, Mainz, Germany; 5 Dr. Senckenberg Institute of Neurooncology, University Hospital Frankfurt, Frankfurt am Main, Germany; 6 University Cancer Center (UCT) Frankfurt-Marburg, University Hospital Frankfurt, Goethe University, Fankfurt am Main, Germany; 7 German Cancer Consortium (DKTK), Partner site Frankfurt/Mainz, A partnership between DKFZ and University Hospital Frankfurt, Frankfurt am Main, Germany; 8 Department of Neurosurgery, University Hospital Würzburg, Würzburg, Germany; 9 Department of Neurosurgery, Charité - University Medical Center Berlin, Berlin, Germany; 10 Department of Neurosurgery, University Hospital Düsseldorf, Düsseldorf, Germany; 11 Department of Neurosurgery, Klinikum Stuttgart, Katharinenhospital, Stuttgart, Germany; 12 Department of Neurosurgery, Klinikum Barmherzige Brueder Trier, Trier, Germany; 13 Department of Neurosurgery, University Hospital Leipzig, Leipzig, Germany; 14 Department of Neurosurgery, University Medical Center Mainz, Mainz, Germany; 15 Department of Neurosurgery, University Hospital Ulm, Ulm, Germany; 16 Center for Neurosurgery, University Hospital Cologne, Cologne, Germany; 17 Department of Neurology, Klinikum Ludwigsburg, Ludwigsburg, Germany; 18 Department of Neurology, Klinikum rechts der Isar, Technical University Munich, Munich, Germany; 19 Department of Neurosurgery, Klinikum rechts der Isar, Technical University Munich, Munich, Germany; 20 University Center for Tumor Diseases (UCT), University Medical Center Mainz, Mainz, Germany; 21 Department of Neurosurgery, Eberhard Karls University Tübingen, Hoppe-Seyler Strasse 3, 72076 Tübingen, Germany

**Keywords:** Distress, Psychosocial burden, Psychosocial care, Supportive care needs, Assessment, Screening, Primary brain tumor patients, High-grade glioma

## Abstract

**Purpose:**

Patients diagnosed with high-grade gliomas (HGG) often experience substantial psychosocial dis-tress. However, due to neurological and neurocognitive deficits its assessment remains challenging, and needs remain unmet. We compared a novel face-to-face assessment during doctor-patient conversations with questionnaire-based screening.

**Methods:**

In this multicenter, two-arm cluster-randomized study involving 13 centers patients in the interven-tion group (IG) were screened for distress via physician-patient conversations, while the control group (CG) completed the Distress Thermometer. Primary outcome was the proportion of patients with poor emotional functioning (measured with the EORTC Quality of Life Questionnaire) who received specialized psychosocial care (PC) within 3 months. Data were collected via patient and physician reports and medical records. Analysis employed mixed models logistic regression.

**Results:**

In total, 763 patients were enrolled at baseline, and 506 completed the follow-up. The emotional functioning was poor in 302/506 (59.7%). The frequency of patients reporting PC utilization was comparable between groups (IG 93/168, 55.4% vs. CG 87/134, 64.9%, odds ratio (OR) =0.67, 95% confidence interval (CI)=0.40-1.11, p=0.115). Likewise, the provision of information about special-ized psycho-oncological care was similar (IG 112/168, 66.7% vs. CG 94/134, 70.1%, OR=0.95, 95%CI=0.39-2.29, p=0.904).

**Conclusion:**

Physician-led, face-to-face distress screening was not superior to questionnaire-based screening in facilitating psychosocial care referrals. Nonetheless, it represents a feasible and patient-centered alternative, particularly for patients with high-grade gliomas suffering from neurocognitive or func-tional deficits.

**Supplementary Information:**

The online version contains supplementary material available at 10.1007/s11060-025-05223-6.

## Introduction

 With an annual incidence rate of 6/100,000, high-grade glioma represents the most common primary brain tumor [1]. The median overall survival varies significantly. Patients diagnosed with glioblastoma survive approximately 15 months in selected clinical trials populations, whereas patients with oligodendroglioma, Isocitrate dehydrogenase (IDH)- mutant, survive up to 15 years following tumor-specific guideline treatment, which comprises maximum safe resection, radiotherapy, and systemic therapy [2, 3]. 

Despite differences in prognosis, patients with high-grade gliomas experience a substantial symptom burden, including neurological symptoms, leading to neurocognitive deficits and disruptions in role and social functioning. Furthermore, psychiatric symptoms such as depression and anxiety are frequent comorbidities in these patients [4]. Consequently, individuals diagnosed with gliomas require psychosocial support to maintain quality of life [5]. While guidelines recommend the assessment of unmet needs and regular psycho-oncological screening for HGG patients, the implementation of such procedures and the provision of care remain inadequate in clinical practice, despite the significant unmet needs [6–9]. 

In Germany, it is a requirement for neuro-oncological centers certified according to the criteria of the German Cancer Society that patients are screened for psycho-oncological distress. In order to identify cancer patients requiring support, various psychosocial screening instruments, such as the NCCN Distress Thermometer (DT), have been developed. The DT includes a numerical analogue scale to assess distress and a problem list to evaluate support needs. It has been validated for brain tumor patients and is considered a reliable tool for identifying distressed individuals [4, 10, 11]. However, psychosocial screening instruments have seldom been adapted to meet the diverse needs of neuro-oncological patients, e.g. neurocognitive deficits are rarely considered [12]. 

Given that patients with high-grade gliomas often experience early neurocognitive decline as a result of the disease itself and its treatment [13–15]it is crucial to either adapt existing instruments to better meet the needs of neuro-oncological patients or explore alternative assessment approaches. We felt the need to simplify the screening process in the hope of intensifying the doctor-patient consultation and to improve patients’ care.

The aim of the “Glioma patients in outpatient care-optimization of psychosocial care in neuro-oncological patients” (GLIOPT) trial was to investigate whether integrating screening questions into doctor-patient consultations (intervention group, IG) could lead to improved identification and support of patients in need, in comparison to optimal standard care (control group, CG).

## Methods

### Study design

In a cluster-randomized, controlled, non-blinded, multicenter study with two parallel groups, we examined the effects of two different approaches to assess psychosocial distress in outpatient patients diagnosed with HGG. The rationale and study protocol were previously published and is summarized below [16].

Our objective was to determine whether psychosocial assessment in patients with HGG could be improved by face-to-face evaluations during doctor-patient consultations. The primary outcome was the percentage of distressed patients with HGG receiving psychosocial care (as reported by patients and by physicians). Participating centers were randomly assigned to either the intervention group or the standard of care (SOC).

The study teams gave each eligible patient an overview of the study and ask whether he or she would be interested in participation. Informed consent was obtained from all individual participants included in the study. The same patient could be approached to participate only once, but at a flexible timepoint during treatment or follow up.

Eligibility criteria for patients were as follows:

1. Diagnosis of either a glioblastoma or gliosarcoma.
2. Diagnosis of an anaplastic astrocytoma WHO grade III or anaplastic oligodendroglioma WHO grade III (according to WHO 2016 classification [17])
3. Age 18 years or older.
4. Capacity to provide informed consent and understand the questionnaires in German language.

### Data collection

Patients in both trial arms were evaluated before (t1) and after (t2) their patient-doctor consultations, with a follow-up assessment at 3 months (t3) after baseline. Both arms of the study underwent identical assessments, which are comprehensively described in the study protocol [16] and outlined in section “measures” below.

### Intervention and control conditions

In the IG, psychosocial distress was assessed during the patient-doctor consultation using the following three questions, which have been developed beforehand [18] and adapted regarding the wording after a pilot testing:

1. Has your mood worsened due to the disease?
2. Do physical changes due to the disease, such as numbness, weakness or feeling exhausted more quickly, put a strain on you?
3. Has your mental capacity worsened because of the disease, making it harder for you to concentrate or remember things, for example?

In both arms, the provision of care was based on urgency, requiring timely attention within a few days to a maximum of 2 weeks, depending on the capacity of the relevant cooperating services, including psycho-oncological and social-legal support, among others.

In the CG, the DT was assessed before the consultation and the results presented to the treating physician.

### Measures

Before the doctor-patient consultation (t1), patients completed a baseline questionnaire assessing their use of and desire for psychosocial support. The completion was supported by study personnel if required.

This questionnaire was based on an adapted version of a self-developed instrument known as the Patients’ Perspective Questionnaire (PPQ) [19]. Additionally, patients completed the European Organisation of Research and Treatment of Cancer Quality of Life Core Questionnaire (EORTC QLQ-C30) along with its brain module (BN20) [20, 21]. The Emotional Functioning Scale of the EORTC QLQ-C30 was used to define burden. This scale consists of four questions regarding a patient’s emotional burden, which are to be answered on a 4-point Likert scale. The sum of the responses is transformed to values ranging from 0 to 100, with lower values indicating a high emotional distress. Subsequently, the patients were categorized into *burdened* and *non-burdened*, with the cut-off set at 71 points (≤ 71 vs. >71) according to Giesinger et al. [22] The doctors were blinded regarding the results of the EORTC QLQ C30 + BN 20 results.

Patients in the CG additionally completed the NCCN Distress Thermometer (DT), a self-reporting screening tool that measures psychosocial distress using a numerical rating scale [10, 11]. 

Following the doctor-patient consultation (t2), patients completed the support needs questionnaire PPQ once again.

In the follow-up after 3 months (t3), patients once again completed the EORTC QLQ-C30 + BN20 and PPQ.

### Sample size

The sample size calculation has been reported previously in detail [16]. Based on previous studies, we expected that in the control group, only about 15% of distressed patients would receive psychosocial care, compared to an expected 25% in the intervention group. We assumed an intra-cluster correlation coefficient (ICC) of 0.005. Therefore, in at least 12 clinics (clusters), a total of *n* = 616 patients were required to demonstrate the expected intervention effect (25% vs. 15%) with an α of 0.05 and a power of 80%.

### Clusters

Outpatient clinics of thirteen neuro-oncological centers throughout Germany participated in the study, including ten university hospitals (Tuebingen, Mainz, Frankfurt, Wuerzburg, Munich, Cologne, Duesseldorf, Berlin, Ulm, Leipzig), and three tertiary referral hospitals (Ludwigsburg, Stuttgart, Trier). The clinics were randomly allocated to either IG (*n* = 6) or CG (*n* = 7). The randomization did not depend on the university status of the centers, the cluster size was equal in both arms.

### Primary outcome and its operationalization

The term “*specialized psychosocial services”* (psycho-oncological care, PC) encompasses various treatment and counseling settings. In total, 13 items were defined providing information on the offer and utilization of these services, according to the study protocol. Therefore, the following different types of care were defined to operationalize the primary outcome, which were assessed at t3 (follow up).

(1) Specialized psychosocial services in the hospital offered (**patients’ report**).

- Psycho-oncological services.
- Social services.

(2) Specialized psychosocial services in the hospital used (**patients’ report**).

- Psycho-oncological services.
- Social services.

(3) Specialized psychosocial services in the outpatient setting used (**patients’ report**).

- Counselling center.
- Psychologist/psychotherapist.
- Psycho-oncologist.

(4) Non-specialized psychosocial services in the outpatient setting used (**patients’ report**).

- General practitioner.
- Pastoral worker.
- Self-help group.

(5) Patients referred to (**doctors’ report**).

- Psychologist/Psychotherapist.
- General practitioner.
- Oncologist.
- Palliative care team.
- Self-help group.
- Social worker.
- Case manager.
- Health insurance.

(6) Use of psychosocial services during inpatient care (according to **medical records**).

- Psychological service.
- Social service.

If at least one of the items within each “type of care” was answered with “yes,” this type of care was assigned a value of 1; otherwise, it was assigned a value of 0.

Subsequently, the following two composite endpoints were defined:

(I) Information about specialized psychosocial services according to the patient’s (1) or the doctor’s report (5, psychologist/psychotherapist or social worker) at t3.
(II) Use of specialized psycho-social services indicating the patient received either inpatient or outpatient specialized psychosocial services as reported by the patient (2, 3) or as documented by the medical records (6) at t3.

We excluded non-specialized outpatient psychosocial care according patients’ report (4).

### Statistical analysis

To account for the clustered data structure, mixed logistic regression models were fitted. These models consider possible intra-cluster correlation by including a clinic-specific random intercept. We adjusted for sex, age, Karnofsky Performance Status (KPS) and primary versus progressive disease, time since initial diagnosis, partnership status (alone vs. in partnership), and employment status.

To quantify the effect of the intervention, each regression model includes a dummy variable that takes a value of 1 if a patient was in the IG and 0 otherwise. We determined the probability of observing a positive outcome regarding the endpoint between the IG and CG with a Wald-Test.

### Missing data

Given that it is unreasonable to assume that all missing data follow a missing-completely-at-random (MCAR) pattern, we employed multiple imputation to address missing data effectively. These imputations were conducted using the ‘mice’ package in R, resulting in the creation of a total of 20 fully imputed datasets.

The statistical analyses regarding the primary endpoint were independently performed for each imputed dataset. The final estimates of the regression coefficients were then calculated as the means across all imputed datasets. The final standard errors were determined using Rubin’s pooling rules.

## Results

**Fig. 1:**
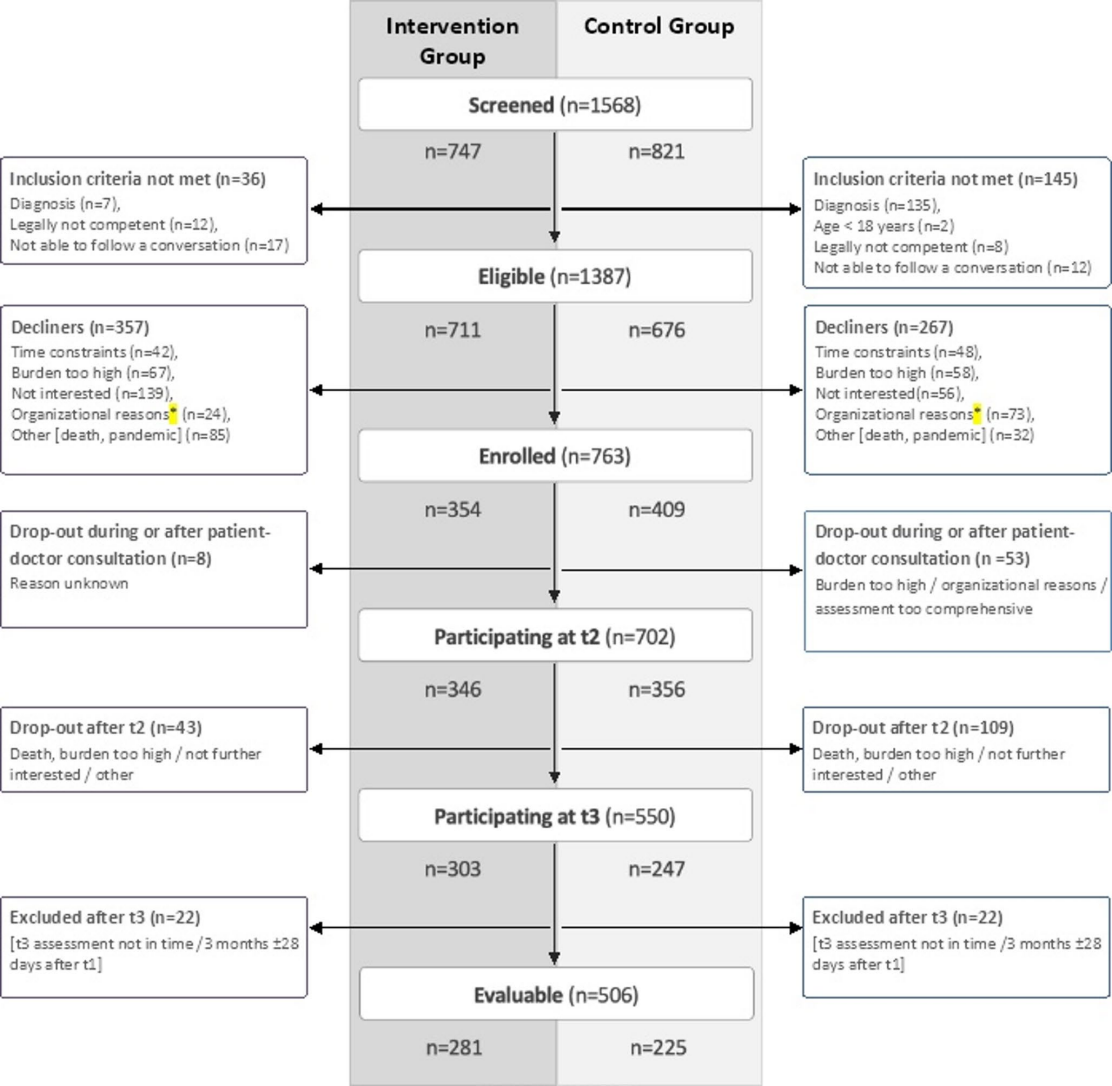
CONSORT diagram adapted for cluster randomized trials. Overview: Screening and enrolment of patients as well as drop-outs are provided separately in the intervention group and the control group of the trial. * e.g. foreseeable that the next appointment will not be scheduled in the timeline of the study, so that the patients were not able to present at the center

The study recruited from 09/2019 to 12/2022. The course of the study is displayed in Fig. 1 (adapted CONSORT diagram for cluster-randomized trials). Due to the COVID-19 pandemic, the recruitment phase, initially planned for 24 months, had to be extended to 39 months. The centers were randomized centrally by the Interdisziplinäres Zentrum für Klinische Studien (IZKS) Mainz. A total of 6 clinics were allocated to the intervention group and 7 clinics to the control group.

### Overall patient sample

A total of 1568 patients with HGG were screened and *n* = 763 were enrolled (354 patients in the IG and 409 in the CG). The reasons for non-participation are described in detail in Fig. 1. The median number of enrolled patients per cluster was 60 (range 19–101).

At baseline (t1) patients’ characteristics were equally distributed in both groups, details are provided in supplementary Table 1. Median age was 54 years (range 19–86), there were more male patients than females (IG: 202, 57.1%, CG: 229, 56.0%). Most of the patients lived with a partner (IG: 253, 71.5%, CG: 277, 67.7%), and most were diagnosed with a glioblastoma (IG: 208, 58.8%, CG: 254, 62.1%).

The study was planned and started in 2019, therefore, the diagnoses are defined according to the WHO classification 2016. However, 18 (2.4%) patients had the diagnosis of an anaplastic oligoastrocytoma according to WHO classification 2007 and it was not possible to retrospectively re-evaluate the diagnosis according to the WHO 2016 classification.

### Patients included in the final outcome analysis

In total, *n* = 506 patients were included in the final analysis, median age was 52 years (range 19–86), 286 were male (56.5%). Median time since diagnosis was 12 months (0.1–309), *n* = 356 had first diagnosis (70.4%) and 53/506 (10.5) patients had progressive disease according to the RANO criteria at t1 (local assessment). Most of the patients were diagnosed with a glioblastoma (294/506, 58.1%; anaplastic astrocytoma WHO grade III: 132/506, 26.1%; oligodendroglioma WHO grade III: 68/506 (13.4%), anaplastic oligoastrocytomas WHO grade III: 12/506, 2.4%). A total of 380/506 patients lived with a partner (75.1%). Details are provided in Table 1, handling of missing data is provided in supplementary Table 2.

**Table 1:** Final analysis cohort, patients’ characteristic at baseline (t1), the groups showed no statistic differences regarding the characteristics (all *p* > 0.05)

Item	Overall (*n* = 506)	Intervention group (*n* = 281)	Control group (*n* = 225)
Age in years (range)	53 (19–86)	52 (19–86)	54 (20–82)
Sex			
Male	286 (56.5%)	160 (56.9%)	126 (56.0%)
Female	219 (43.3%)	121 (43.1%)	98 (43.6%)
Divers	1 (0.2%)	0 (0.0%)	1 (0.4%)
Family situation			
Single	118 (23.3%)	69 (24.6%)	49 (21.8%)
Married	328 (64.8%)	182 (64.8%)	146 (64.9%)
Divorced	39 (7.7%)	22 (7.8%)	17 (7.6%)
Widowed	17 (3.4%)	6 (2.1%)	11 (4.9%)
Missing	4 (0.8%)	2 (0.7%)	2 (0.9%)
Partner			
No	102 (20.2%)	61 (21.7%)	41 (18.2%)
Yes	380 (75.1%)	203 (72.2%)	177 (78.7%)
Missing	24 (4.7%)	17 (6.0%)	7 (3.1%)
Professional qualification			
Training	208 (41.1%)	98 (34.9%)	110 (48.9%)
Technical/Master school	53 (10.5%)	36 (12.8%)	17 (7.6%)
Technical college, engineering school	52 (10.3%)	28 (10.0%)	24 (10.7%)
University	111 (21.9%)	71 (25.3%)	40 (17.8%)
Other	28 (5.5%)	19 (6.8%)	9 (4.0%)
None	27 (5.3%)	15 (5.3%)	12 (5.3%)
Missing	27 (5.3%)	14 (5.0%)	13 (5.8%)
Employment			
At least 50%	205 (40.5%)	112 (39.9%)	93 (41.4%)
Reduction in earning capacity pension	70 (13.8%)	38 (13.5%)	32 (14.2%)
Retirement pension	103 (20.4%)	53 (18.9%)	50 (22.2%)
Other	72 (14.3%)	45 (15.9%)	27 (12.1%)
Missing or unknown	56 (11.1%)	33 (11.7%)	23 (10.3%)
Monthly income			
< 1000 EUR	0 (0%)	0 (0%)	0 (0%)
1001–2000 EUR	77 (15.2%)	40 (14.2%)	37 (16.4%)
2001–3500 EUR	192 (37.9%)	109 (38.8%)	83 (36.9%)
> 3500 EUR	141 (27.9%)	86 (30.6%)	55 (24.4%)
Missing	96 (19.0%)	46 (16.4%)	50 (22.2%)
Diagnosis			
Glioblastoma WHO grade IV	294 (58.1%)	159 (56.6%)	135 (60.0%)
Astrocytoma WHO grade III	132 (26.1%)	78 (27.8%)	54 (24.0%)
Oligodendroglioma WHO grade III	68 (13.4%)	39 (13.9%)	29 (12.9%)
Oligoastrocytoma WHO grade III (according to WHO 2007 classification)	12 (2.4%)	5 (1.8%)	7 (3.1%)
Stage of disease			
First diagnosis	356 (70.4%)	192 (68.3%)	164 (72.9%)
Progression	150 (29.6%)	89 (31.7%)	61 (27.1%)
Time since diagnosis in months			
Median (range)	12 (0.1–309)	13 (0.5–287)	11 (0.1–309)
Missing	7 (1.38%)	3 (1.07%)	4 (1.78%)
Situation (MRI) according to RANO criteria at t1			
Complete response	108 (21.3%)	55 (19.6%)	53 (23.6%)
Partial response	56 (11.1%)	32 (11.4%)	24 (10.7%)
Stable disease	285 (56.3%)	164 (58.4%)	121 (53.8%)
Progressive disease	53 (10.5%)	29 (10.3%)	24 (10.7%)
Missing	4 (0.8%)	1 (0.4%)	3 (1.3%)
KPS at t1			
Median (range)	90 (40–100)	90 (40–100)	90 (40–100)
< 70%	25 (4.9%)	12 (4.3%)	13 (5.8%)
≥70%	480 (94.9%)	268 (95.4%)	212 (94.2%)
Missing	1 (0.2%)	1 (0.4%)	0 (0.0%)

### High psychosocial burden in both groups

At t1, emotional functioning was low both in the entire sample (mean EF score 59.9, SD 28.1) and in the two arms (IG: mean 59.4, SD 28.8; CG: 60.6, SD 27.3). Clinically relevant burden (EF score ≤71) was present in 302/506 (59.5%). This was similar in the IG (168/281, 60.0%) and CG (134/225, 59.8%). At t3, the EF scores were comparable; details are provided in Table 2.

**Table 2:** Clinically relevant burdened patients in interventions group (IG)/control group (CG) at t1 and t3 according to the emotional functioning (EF) scale with cut-off according to Giesinger et al. [22]

Emotional functioning according to EORTC QLQ-C30	Overall, *n* = 506	IG, *n* = 281	CG, *n* = 225
t1			
Mean (SD, range)	59.9 (28.1, 0-100)	59.4 (28.8, 0-100)	60.6 (27.3, 0-100)
EF score > 71	202 (39.9%)	112 (39.9%)	90 (40.0%)
EF score ≤71	302 (59.7%)	168 (59.8%)	134 (59.6%)
Missing	2 (0.4%)	1 (0.4%)	1 (0.4%)
t3			
Mean (SD, range)	62.6 (27.7, 0-100)	61.8 (28.1, 0-100)	63.8 (27.3, 0-100)
EF score > 71	204 (40.3%)	109 (38.8%)	95 (42.2%)
EF score ≤71	296 (58.5%)	168 (59.8%)	128 (56.9%)
Missing	6 (1.2%)	4 (1.4%)	2 (0.9%)
Change Score (t3-t1)			
Mean (SD)	2.63 (24.1)	2.37 (25.5)	2.95 (22.3)
Unknown	8	5	3

The groups showed no statistic differences regarding the characteristics (all *p* > 0.05)

### Doctor patient consultation and psychosocial care provided and used was comparable in the IG and the CG

Doctor patient consultation was similar in length in both groups (IG: 15–26 min, mean = 23.06 min, SD = 9.16 and CG :12–35 min, mean = 23.04 min, SD = 14.63). Most patients (71%) reported feeling relieved after the consultation, no significant difference between IG and CG (Fisher"s exact test, p = 0.322).

The proportion of highly distressed patients who were informed about PC (IG 93/168, 55.4%, CG 87/134, 64.9%,) and the proportion of those who used these services (IG 112/168, 66.7%, CG 94/134, 70.1%) were higher in the CG without being significant. We found no evidence, that the three screening questions led to higher information about PC (OR = 0.95, 95%CI = 0.39–2.29, *p* = 0.904) or use of PC in the IG compared to CG (OR = 0.67, 95%CI = 0.40–1.11, *p* = 0.115). Details are provided in Table 3.

**Table 3:** Primary outcome in intervention group (IG) and control group (CG)

	Care provided/used per group	Odds Ratio	95% CI	*P*-Value
Type of care				
(1) Specialized psychosocial services in the hospital offered	IG 108/168 (64.3%) vs. CG 91/134 (67.9%)	0.93	0.39–2.21	0.864
(2) Specialized psychosocial services in the hospital used	IG 62/168 (36.9%) vs. CG 61/134 (45.5%)	0.71	0.38–1.30	0.261
(3) Specialized psychosocial care in the outpatient setting used	IG 60/168 (35.7%) vs. CG 50/134 (37.3%)	0.81	0.49–1.36	0.424
(4) Non-Specialized psychosocial care in the outpatient setting used	IG 35/168 (20.8%) vs. CG 26/134 (19.4%)	0.81	0.47–1.41	0.463
(5) Patients referred (according to doctors’ report)	IG 46/168 (27.4%) vs. CG 30/134 (22.4%)	1.26	0.61–2.59	0.526
(6) Use of psychosocial services during inpatient care (according to medical records)	IG 50/168 (29.8%) vs. CG 29/134 (21.6%)	1.24	0.47–3.29	0.671
Primary endpoint				
(I) Information about specialized psycho-oncological care	IG 112/168 (66.7%) vs. CG 94/134 (70.1%)	0.95	0.39–2.29	0.904
(II) Use of specialized psycho-oncological care	IG 93/168 (55.4%) vs. CG 87/134 (64.9%)	0.67	0.40–1.11	0.115

Inpatient care provided (patients’ report) was comparable in both groups. Inpatient care used (patients’ report) was reported more frequently in the CG than in the IG. In the IG, patients made more frequently use of outpatient psychosocial care. The use of outpatient care by general practitioners, pastoral workers, or self-help groups (patients’ report) was equal in both groups. The doctors in the IG reported that they referred burdened patients slightly more frequently to psychologist, psychotherapist, general practitioner, oncologist, social services, palliative care team, self-help group in 27.4% of the cases vs. 22.4% in the CG (OR = 1.26, 95%CI = 0.61–2.59, *p* = 0.56). According to medical records more patients in the IG (29.8%) made use of psychosocial services than in the CG (21.6%, OR = 1.24, 95%CI = 0.47–3.29, *p* = 0.671).

### Adherence to protocol

Compliance to the psychosocial assessment according to the study protocol was observed more frequently in the IG using three questions than in the CG by the DT (IG: 353/354, 99.7% vs. CG 388/409, 94.9%, *p* = 0.005, OR = 19).

In the IG more decliners of participation were observed than in the CG (50.2% vs. 39.5%), whereas more drop-outs occurred in the CG than in the IG (39.6% vs. 14.4%). In total, 213/763 patients dropped out (27.9%). In another 44 patients, the t3 assessment was performed outside of the time window per protocol and therefore not included in the final analysis, see also Fig. 1.

## Discussion

In this prospective cluster-randomized multicenter study we demonstrated that screening questions implemented in the patient-doctor consultation were feasible in clinical routine and resulted in a similar proportion of psychosocial healthcare provision for patients in need compared to validated questionnaires.

The patient sample reflects the sociodemographic profile of patients with HGG, with a higher representation of males than females and the majority being diagnosed with glioblastoma. Both study groups showed similar sociodemographic characteristics. The proportion of patients in need aligns with data reported by others, underscoring the consistent burden experienced by patients with HGG throughout their disease trajectory [6, 8, 23]. Furthermore, during the development of the screening questions conducted in a comparable patient population encompassing all disease stages, we observed no differences regarding relevant topics covered in the subpopulations of patients with first diagnosis and patients with tumor progression [18]. Therefore, these questions may be applicable across all disease stages. The study was planned and initiated in 2019, and consequently, diagnoses were defined in accordance with the WHO classification from 2007 to 2016 [17, 24]. However, previous research conducted by our group and others has indicated that the psychological burden of patients diagnosed with a glioma is largely independent of histology [13, 25]. The number of included patients varied across the clusters, calling for a cautious interpretation of the data. However, cluster randomization facilitated the conduct of the study and the enrollment of patients because the clinical procedures could remain the same within each center.

The notably high psychological burden with poor emotional functioning in 302 out of 506 patients (59.7%) was comparable in both study groups, consistent with previous findings and emphasizes the need of screening methods easily integrable in clinical routine [26]. 

In our study, the integration of the screening into the doctor-patient consultation resulted in a comparable identification of patients in need of psychosocial support. Therefore, this novel approach utilizing the three questions face-to-face may facilitate screening in patients unable to complete a questionnaire.

Considering the slightly higher frequency of patients being referred to social services based on doctors’ responses and medical records in the intervention group, it could be assumed that doctors in this group were more aware to identify unmet needs. The screening questions might have deepened the exploration of the doctors, although the consultation time was similar in both groups. Despite the proportion of patients receiving PC in the intervention group was not higher than in the control group, the reintroduction of psychosocial issues into doctor-patient-conversations was feasible.

Yet, referral to PC depends not only on screening procedures but also on capacities of an individual center, which were already largely used. As has been noted by others, the implementation of screening procedures often encounters challenges in the presence of department structures and resource constraints [26]. Therefore, our results show that there is a certain lack of capacity in PC leading to lower referral and utilization rates. Furthermore, it is important to acknowledge that not all patients experiencing psychosocial burden necessarily desire or are willing to receive support, a phenomenon reported by others [27, 28]. Therefore, burdened patients may decline support independently of the screening procedure.

Due to the COVID-19 pandemic, the enrolment phase had to be extended because patients were more hesitant to participate in studies. Furthermore, it led to a considerable dropout. This may have negatively influenced our results because psychological burden and unmet needs may have been more pronounced whereas at the same time PC resources were limited [29]. It is worth noting that high dropouts, especially in non-AMG or MPG studies, have been reported by other researchers as well [30].

This may have introduced a selection bias, as patients experiencing clinical deterioration may have been more prone to drop out, potentially leading to their underrepresentation in the study [31].

The fact that most of the clusters were university hospitals and all of them certified Centers of Neuro-Oncology presumably positively influenced the proportion of patients in need and the provision of care as the certification requirements include psychosocial screening. Nevertheless, the comparison between intervention and control group would still be valid.

Finally, the intervention was well-received and accepted. This is emphasized by the fact, that the protocol adherence in the IG was significantly higher than in the CG. We therefore conclude that the three questions were not only well implementable in the doctor-patient consultation but also represent a novel approach for a patient-centered assessment in neuro-oncological patients. Results of this study may contribute to streamlining and improving screening procedures in this specific patient cohort.

### Strengths and limitations of the study

This is one of the few randomized controlled studies comparing different screening procedures for patients diagnosed with HGG. Due to low-threshold inclusion criteria and cluster randomization leading to a “single-arm structure” of the study per center, patients who often do not meet the inclusion criteria were able to participate in this trial. The screening questions for the doctor-patient conversation can be implemented by neuro-oncologists, radio-oncologists, general practitioners, and all other participating disciplines to improve their doctor-patient consultations. This allows for a psychosocial assessment even if no screening by a validated questionnaire is feasible. The randomized design, application of standard operating procedures, and targeted training of study personnel represent a strength of this trial.

Of note, the following limitations must be considered: The pandemic situation and a considerable decliner rate and drop-out might have led to a selection bias. At the same time, the pandemic might also have had an impact on psychosocial care provision and the pandemic related stress on patients, families, interpersonal interactions, and psychosocial providers may have significantly influenced the outcomes of this study, which might limit the generalizability of the results.

Furthermore, it is important to acknowledge that due to the high median KPS score (90) and a significant proportion of patients with stable disease, there may be an underestimation of psychological burden and unmet needs (survival bias). Another limitation is, that we did not assess qualitative data which would have been helpful to evaluate the deepness of the discussion in the consultations as this impacts sometimes more than the support. Furthermore, due to the methodological approach, resource utilisation efficiency and the appropriateness of psychosocial care provided were not evaluated. Medical providers might also have been hesitant to refer to PC if resources were limited in a certain center, which is a confounding factor and a disadvantage of the cluster randomization. However, the authors doubt if this fact could have been eliminated by traditional randomization. We did not perform neurocognitive screening tests at the beginning, which would have provided important information about the patient population regarding ability to complete questionnaires. However, as assistance with completing the questionnaires was allowed, we were able to include neurocognitive impaired patients and therefore, specific assessment in our opinion was not necessary.

The psycho-emotional burden as per EORTC-QLQ C30 was used as the basis for the primary outcome. The emotional functioning did not change significantly between t1 and t3. This could be (falsely) interpreted as lack of efficiency of the support systems in this specific context.

Finally, the patient population in our study was heterogeneous in terms of diagnosis, disease trajectory and status of the disease and socioeconomic factors. This might also impact the generalizability of the study findings. At the same time, this may also represent a strength of the study, as we successfully applied the questions to patients in a wide variety of clinical situations.

## Conclusion

Physician-led, face-to-face distress screening was not superior to questionnaire-based screening in facilitating psychosocial care referrals. Nonetheless, it represents a feasible and patient-centered alternative, particularly for patients with high-grade gliomas suffering from neurocognitive or functional deficits.

## Supplementary Information

Below is the link to the electronic supplementary material.

Supplementary Material 1

## Data Availability

No datasets were generated or analysed during the current study.

## Declarations

The study has been registered in the German Clinical Trials Register, registration number: DRKS00018079. Protocol Version: Version 4.0 (01/11/2018).
