# Supplementary material for: Physician-led versus questionnaire-based psychosocial screening in adults with high-grade glioma: a cluster-randomized controlled trial (GLIOPT)
Source: J Neurooncol. 2025 Sep 10;175(3):967–77. doi: 10.1007/s11060-025-05223-6 (PMC12511251; doi:10.1007/s11060-025-05223-6)
Supplement: Supplementary file 1 — Supplementary Material 1 [file 11060_2025_5223_MOESM1_ESM.docx]

**Supplementary material**

**Supplementary table 1: Overall enrolled patients’ characteristics**

| **Item** | **Overall**  **(n=763)** | **Intervention group**  **(n=354)** | **Control group**  **(n=409)** |
| --- | --- | --- | --- |
| **Age in years (range)** | 54 (19-86) | 53 (19- 86) | 55 (20-84) |
| **Sex** | | | |
| Male | 431 (56.5%) | 202 (57.1%) | 229 (56.0%) |
| Female | 331 (43.4%) | 152 (42.9%) | 179 (43.8%) |
| Divers | 1 (0.1%) | 0 (0.0%) | 1 (0.2%) |
| **Family situation** | | | |
| Single | 153 (20.1%) | 80 (22.6%) | 73 (17.8%) |
| Married | 453 (59.4%) | 226 (63.8%) | 227 (55.5%) |
| Divorced | 61 (8.0%) | 30 (8.5%) | 31 (7.6%) |
| Widowed | 27 (3.5%) | 8 (2.3%) | 19 (4.6%) |
| Missing | 69 (9.0%) | 10 (2.8%) | 59 (14.4%) |
| Partner | | | |
| No | 137 (18.0%) | 73 (20.6%) | 64 (15.6%) |
| Yes | 530 (69.5%) | 253 (71.5%) | 277 (67.7%) |
| Missing | 96 (12.6%) | 28 (7.9%) | 68 (16.6%) |
| **Professional qualification** | | | |
| Training | 285 (37.4%) | 124 (35.0%) | 161 (39.4%) |
| Technical / Master school | 73 (9.6%) | 45 (12.7%) | 28 (6.8%) |
| Technical college, engineering school | 71 (9.3%) | 33 (9.3%) | 38 (9.3%) |
| University | 160 (21.0%) | 88 (24.9%) | 72 (17.6%) |
| Other | 37 (4.8%) | 21 (5.9%) | 16 (3.9%) |
| None | 35 (4.6%) | 17 (4.8%) | 18 (4.4%) |
| Missing | 102 (13.4%) | 26 (7.3%) | 76 (18.6%) |
| **Employment** | | | |
| At least 50% | 291 (38.2%) | 138 (39.0%) | 153 (37.4%) |
| Reduction in earning capacity pension | 94 (12.3) | 43 (12.1%) | 51 (12.5%) |
| Retirement pension | 144 (18.9%) | 67 (18.9%) | 77 (18.8%) |
| Other | 97 (12.7%) | 57 (16.2%) | 40 (9.8%) |
| Missing or unknown | 137 (17.9%) | 49 (13.8%) | 88 (21.5%) |
| **Monthly income** | | | |
| ≤1000 EUR | 40 (5.2%) | 20 (5.6%) | 20 (4.9%) |
| 1001-2000 EUR | 105 (13.8%) | 48 (13.6%) | 57 (13.9%) |
| 2001-3500 EUR | 254 (33.3%) | 138 (39.0%) | 116 (28.4%) |
| >3500 EUR | 190 (24.9%) | 100 (28.2%) | 90 (22.0%) |
| Missing | 174 (22.8%) | 48 (13.6%) | 126 (30.8%) |
| **Diagnosis** | | | |
| Glioblastoma, WHO grade IV | 462 (60.6%) | 208 (58.8%) | 254 (62.1%) |
| Astrocytoma WHO grade III | 184 (24.1%) | 92 (26.0%) | 92 (22.5%) |
| Oligodendroglioma WHO grade III | 94 (12.3%) | 46 (13.0%) | 48 (11.7%) |
| Oligoastrocytoma WHO grade III (according to WHO 2007 classification) | 18 (2.4%) | 8 (2.3%) | 10 (2.4%) |
| Other | 5 (0.7%) | 0 (0%) | 5 (1.2%) |
| **Stage of disease** | | | |
| First diagnosis | 538 (70.5%) | 244 (68.9%) | 294 (71.9%) |
| Progression | 224 (29.4%) | 110 (31.1%) | 114 (27.9%) |
| Missing | 1 (0.1%) | 0 (0%) | 1 (0.2%) |
| **Time since diagnosis in months** | | | |
| Median (range) | 12 (0 - 309) | 13 (0-288) | 11 (0-309) |
| Missing | 13 (1.70%) | 5 (1.41%) | 8 (1.96%) |
| **Situation (MRI) according to RANO criteria at t1** | | | |
| Complete response | 138 (18.1%) | 66 (18.6%) | 72 (17.6%) |
| Partial response | 85 (11.1%) | 34 (9.6%) | 51 (12.5%) |
| Stable disease | 419 (54.9%) | 203 (57.3%) | 216 (52.8%) |
| Progressive disease | 115 (15.1%) | 50 (14.1%) | 65 (15.9%) |
| Missing | 6 (0.8%) | 1 (0.3%) | 5 (1.2%) |
| **KPS at t1** | | | |
| Median (range) | 90 (40-100) | 90 (40-100) | 90 (40-100) |
| <70% | 54 (7.1%) | 19 (5.4%) | 35 (8.6%) |
| ≥70% | 704 (92.3%) | 332 (93.8%) | 372 (91.0%) |
| missing | 5 (0.7%) | 3 (0.8%) | 2 (0.5%) |

**Supplementary Table 2: Number of observed values, and number and proportion of missing values, for each variable included in the types of care and primary endpoints.**

Observed and Missing Values by Variable (n = 763)

| **Variable** | **Observed (n)** | **Missing (n)** | **Missing (%)** |
| --- | --- | --- | --- |
| Psycho-oncological services offered up to t3 (patient report) | 533 | 230 | 30.1% |
| Social services offered up to t3 (patient report) | 528 | 235 | 30.8% |
| Counseling center used up to t3 (patient report) | 523 | 240 | 31.5% |
| Psychologist/psychotherapist used up to t3 (patient report) | 521 | 242 | 31.7% |
| Psycho-oncologist used up to t3 (patient report) | 502 | 261 | 34.2% |
| Counseling center used up to t3 (patient report) | 502 | 261 | 34.2% |
| Psychologist/psychotherapist used up to t3 (patient report) | 509 | 254 | 33.3% |
| Counseling by general practitioner used up to t3 (patient report) | 505 | 258 | 33.8% |
| Pastoral worker used up to t3 (patient report) | 501 | 262 | 34.3% |
| Self-help group used up to t3 (patient report) | 503 | 260 | 34.1% |
| Psychological service used up to t3 (medical records) | 763 | 0 | 0.0% |
| Social service used up to t3 (medical records) | 763 | 0 | 0.0% |
| Patient referred to ... (t1, doctor report) | 724 | 39 | 5.1% |

Handling of missing data

Missing data were imputed using a single imputation model comprising univariate conditional models for each incomplete variable. To account for the cluster-randomized study design, multilevel models with clinic-specific random intercepts were included. In total, the imputation model contained 116 variables.

Imputations covered all variables needed for the regression models underlying Table 3, including those defining the six types of care and the two primary endpoints (e.g., binary indicators of psycho-oncological or psychosocial service use up to t3). All seven confounders from the regression analyses and the emotional functioning (EF) scale at t1 were also included, as all models were fitted on patients identified as emotionally burdened at baseline. Variables closely related to those of interest, such as service use up to t1, were incorporated to improve imputations for corresponding t3 variables.

An “impute-then-transform” strategy was applied: when composite scores (e.g., EF) required imputation, their components were imputed first, and the scores were then calculated within each imputed dataset.

To enhance imputation quality, variables with a very high proportion of missing values were excluded as predictors, and models with weakly related predictors were avoided. The quickpred() function restricted predictors in each univariate model to those with ≥ 60% observed values and a Spearman correlation ≥ 0.15 with the target. Under these criteria, each model included about 25 predictors—consistent with van Buuren’s recommendations for balancing imputation bias and variance.

Supplementary table 2 summarizes for all relevant variables the information on the observed and missing values before imputation.
